# Phenolic composition, antioxidant activity, anticholinesterase potential and modulatory effects of aqueous extracts of some seaweeds on β-amyloid aggregation and disaggregation

**DOI:** 10.1080/13880209.2019.1634741

**Published:** 2019-07-23

**Authors:** Tosin A. Olasehinde, Ademola O. Olaniran, Anthony I. Okoh

**Affiliations:** a Department of Biochemistry and Microbiology, Applied and Environmental Microbiology Research Group (AEMREG), University of Fort Hare, Alice, South Africa;; b SAMRC Microbial Water Quality Monitoring Centre, University of Fort Hare, Alice, South Africa;; c Food Technology Department, Nutrition and Toxicology Division, Federal Institute of Industrial Research Oshodi, Lagos, Nigeria;; d Discipline of Microbiology, School of Life Sciences, College of Agriculture, Engineering and Science, University of Kwazulu-Natal, Durban, South Africa

**Keywords:** Alzheimer’s disease, cholinesterases, β-amyloid peptide

## Abstract

**Context:** Seaweeds contain bioactive compounds with different biological activities. They are used as functional ingredients for the development of therapeutic agents to combat degenerative diseases.

**Objective:** This study investigated the phenolic composition, antioxidant activity, cholinesterase inhibitory and anti-amyloidogenic activities of aqueous extracts of *Gracilaria beckeri* (J.Agardh) Papenfuss (Gracilariaceae) (RED-AQ), *Ecklonia maxima* (Osbeck) Papenfuss (Lessoniaceae) (ECK-AQ), *Ulva rigida* (C.Agardh) Linnaeus (Ulvaceae) (URL-AQ) and *Gelidium pristoides* (Turner) Kützing (Gelidiaceae) (GEL-AQ).

**Materials and methods:** Phenolic composition of the seaweed extracts was determined using liquid chromatography mass spectrometry. Radical scavenging and metal chelating activities were assessed *in vitro*. The effect of the extracts (21–84 µg/mL) on acetylcholinesterase and butyrylcholinesterase activities were also investigated using an *in vitro* colorimetric assay. Transmission electron microscope and thioflavin-T fluorescence assay were used to examine the anti-amyloidogenic activities of the extracts.

**Results:** Phloroglucinol, catechin, epicatechin 3-glucoside were identified in the extracts. ECK-AQ (IC_50_=30.42 and 280.47 µg/mL) exhibited the highest OH^•^ scavenging and metal chelating activities, while RED-AQ (41.23 and 334.45 µg/mL) exhibited the lowest. Similarly, ECK-AQ (IC_50_ = 49.41 and 52.11 µg/mL) exhibited higher inhibitory effects on acetylcholinesterase and butyrylcholinesterase activities, while RED-AQ (64.56 and 63.03 µg/mL) showed the least activities. Rapid formation of β-amyloid (Aβ_1-42_) fibrils and aggregates was observed in electron micrographs of the control after 72 and 96 h. The reduction of Aβ_1-42_ aggregates occurred after co-treatment with the seaweed extracts.

**Discussion and conclusion:** ECK-AQ, GEL-AQ, URL-AQ and RED-AQ may possess neuroprotective potential and could be explored for the management of Alzheimer’s disease.

## Introduction

Alzheimer’s disease is a neurological disorder which affects millions of aged individuals across the world (Li et al. 2018). It is the most common kind of dementia that is characterized by redox imbalance in the neurons, cholinergic deficit, formation of neurotoxic amyloid senile plaques, neuroinflammation and neurodegeneration which leads to cognitive decline, learning problems and memory loss (Feng and Wang 2012; Mendiola-Precoma et al. 2016). Although the pathogenesis of AD is complex, development of this neurological disease has been linked with impaired cholinergic pathway which is caused by upregulation of acetylcholinesterase and butyrylcholinesterase as well as rapid depletion of acetylcholine (Adefegha et al. 2016; Ferreira-Vieira et al. 2016). The disruption in the amyloid precursor protein processing pathway has been identified as one of the pathological hallmarks of AD (Singh et al. 2016). Elevated concentrations and/or activity of the β-site amyloid precursor protein-cleaving enzyme in brain cells trigger the formation of β-amyloid peptides which accumulates and aggregate to form senile plaques around the neurons (O'Brien and Wong 2011; Olasehinde, Odjadjare, et al. 2019) . The senile plaques consist of peptides alongside with redox metals such as Zn, Fe^2+^ or Cu which are capable of initiating the production of free radicals and redox imbalance in the neurons (Cheignon et al. 2018). Some of the free radicals produced such as hydroxyl radicals are able to induce oxidative damage to the neurons which leads to cell death and ultimately progression of AD (Olasehinde et al. 2017; Oboh, Ademosun, et al. 2018a). Though cholinesterase inhibitors such as galathanmine, rivastigmine and prostigmine are preferred drugs used for the management of AD, these drugs are able to mitigate cholinergic deficit and cannot prevent or halt the progression of AD. Furthermore, β-amyloid aggregation inhibitors and clearance activators are still under clinical trials. Hence, there has been a great interest in the search for an alternative therapeutic approach for the treatment and/or management of AD.

Seaweeds contain biologically active compounds with several therapeutic potentials (Khalid et al. 2018). They have also found application in food and pharmaceuticals industries as sources of functional food ingredients, nutraceuticals, dietary supplements, agar, food hydrocolloids and drugs (Plaza et al. 2008; Bixler and Porse 2011; Lordan et al. 2011). Some seaweeds are consumed traditionally as soups and vegetables, while others are employed as seasoning and used in sauces. Previous reports have shown their antidiabetic, antihypertensive, antioxidant, anti-inflammatory and immunomodulatory activities (Mohamed et al. 2012; Admassu et al. 2018). However, their neuroprotective effects have not been fully explored. In this study, we evaluated the neuroprotective potential of some selected seaweeds *Gracilaria beckeri* (J.Agardh) Papenfuss (Gracilariaceae), *Ecklonia maxima* (Osbeck) Papenfuss (Lessoniaceae), *Ulva rigida* (C.Agardh) Linnaeus (Ulvaceae) and *Gelidium pristoides* (Turner) Kützing (Gelidiaceae) via their radical scavenging and metal chelating activities as well as their modulatory effects on cholinesterase activities and β-amyloid aggregation and disaggregation.

## Materials and methods

### Materials

Acetylcholinesterase, butyrylcholine iodide, butyrylcholinesterase, acetylcholine iodide, β-amyloid protein (Aβ_1-42_) acetylthiocholine iodide, 5,5-dithiobis-(2-nitrobenzoic) acid, glycine and sodium hydroxide were sourced from Sigma-Aldrich (St. Louis, MO, USA), while thioflavin-T was obtained from Sigma-Aldrich (India).

### Sample collection and identification

*Gelidium pristoides* (GEL-AQ), *Gracilaria beckeri* (RED-AQ) and *Ulva rigida* (URL-AQ) were collected in June 2017 from Port Alfred in Eastern Cape, South Africa. *Ecklonia maxima* (ECK-AQ) was sourced from Kelpak Products (Pty) Ltd. (Cape Town, South Africa). The seaweeds were identified by Dr. Paul-Steyn Pierre at the Department of Botany, Nelson Mandela University, South Africa.

### Aqueous extraction

Seaweeds were washed and allowed to dry at room temperature. Each sample was ground into powder which was soaked in water (1:2 *w/v*) for 24 h at 25 °C. Then, the extract was filtered, and the filtrate obtained was lyophilized and stored in different vials at 4 °C for further analysis.

### UHPLC-ESI-QTOF-MS analysis

Analysis of the phytochemicals present in the seaweed extracts was determined following the method of Kalinski et al. (2019) with slight modification. The UHPLC (Thermo Fisher Scientific, Sunnyvale, CA, USA) used in this study was equipped with C18 (2.1 × 100 mm, 2.2 μm) (Acclaim RSLC 120). The flow rate was set at 0.300 mL/min using mixtures of water and acetonitrile, containing 0.1% formic acid (FA). The analysis was done in LC-MS/MS mode, and the MS analyses were performed on a Bruker Compact QToF mass spectrometer using an electrospray ionization probe (Bruker, Bremen, Germany). The mobile phase consists of water (A) and acetonitrile (B) with 0.1% formic acid and was set to follow a step gradient which includes 90% A and 10% B (0–5 min), 60% A and 40% B (5–15 min), 60% A and 40% B (15–20 min), 30% A and 70% B (20–25 min) 30% A and 70% B (25–30), 100% B (30–35) and 100% (35–40 min). Minimum intensity of the MS was set at 5000 counts, collision energy 40 eV with five precursors. The data obtained were converted to mzXML format using Bruker Compass Software (Bruker, MA, USA). The files generated were subjected to MZmine 2 (2.36 version). Masses were detected from the raw data using the mass detection module, and noise level was set at 15.00. Then, the chromatogram builder icon was used to build a chromatogram for each mass. The minimum timespan (retention time) was set at 0.030, while the minimum height (peak) was set at 25.000. The *m/z* tolerance was set at 0.04 Da or 5.0 ppm. After the peaks were obtained, local minimum search algorithm was used for chromatogram deconvolution. The threshold was set at 65%, while the minimum relative height was 5.0%. Minimum retention time, relative height, absolute height and ration of peak were set at 0.030 min, 5.0%, 50.00 and 2, respectively. The peak duration range was also set between 0.00 and 2.00 min. After this, isotope grouping was done to search for peak lists within the peak with same isotope patterns using the isotope grouper. The peaks were then filtered and de-isotoped. The peaks were identified using the custom search database module; *m/z* tolerance was set at 0.04 Da or 5.0 ppm, while retention time tolerance was set at 0.07 min. The search online database was used to search for similar identities using different online databases on MZmine including PubChem, Kebb, metaCyc and Hmdb.

#### 2,2-azinobis 3-ethylbenzothiazoline-6-sulfonate (ABTS) radical scavenging assay

ABTS radical scavenging activity was assessed using the method of Re et al. (1999). Percentage scavenging activity was calculated using Equation 1:

(1) $$\left(\%\right)\;\text{scavenging}\;\text{activity}=\left({\text{OD}}_{\text{ref}}-{\;\text{OD}}_{\text{sea}}\right)/{\text{OD}}_{\text{ref}}\times 100$$

where OD_ref_ is the optical density of the control experiment, and OD_sea_ is the optical density of the test solution containing extract.

#### 2,2-Diphenyl-1-picrylhydrazyl (DPPH) radical scavenging assay

The method established by Gyamfi et al. (1999) was used to determine the ability of the seaweed extracts to scavenge DPPH^•^. Percentage scavenging activity was calculated using Equation 1.

### Hydroxyl radical scavenging assay

The ability of seaweed extracts to scavenge OH^•^ was investigated following the method established by Halliwell and Gutteridge (1981). Percentage scavenging activity was calculated using Equation 1.

### Fe^2+^ chelation assay

Fe^2+^-chelating activity of the extracts was assessed as reported by Puntel et al. (2005). Percentage chelating ability was obtained using Equation 1.

### Determination of modulatory effects on AChE and BChE

AChE (0.28 U/mL) was prepared in phosphate buffer, and an aliquot (40 µL) was added to a solution containing 5,5-dithiobis-(2-nitrobenzoic) acid, phosphate buffer (0.1 M, pH 8.0, 80 µL) and extracts (21–84 µg/mL) in a 96-well plate (Perry et al. 2000). After 20 min, acetylcholine iodide was added to each well and optical density of the solution was measured at 412 nm using a plate reader. The method was used for the determination of BChE activity. Butyrylcholine iodide was used as the substrate. Percentage inhibition was obtained using Equation 2:

(2) $$\left(\%\right)\;\text{Inhibition}=\left({\text{OD}}_{\text{con}}-{\text{OD}}_{\text{sam}}\right)/{\text{OD}}_{\text{con}}\times 100$$

### Aβ_1-42_ aggregation assay

Aβ_1-42_ was dissolved in sodium hydroxide (50 mM, 200 µL), and 900 µL of deionized water was added to the solution after 3 min. Phosphate-buffered saline (100 µL) was added to the solution to reach a final concentration of 25 µM of peptide. Sonication was done for about 3 min before the mixture was centrifuged for 20 min at 4000×*g* and 4 °C. Aβ_1-42_ (100 µL) (Rosensweig et al. 2012) was incubated with seaweed extracts (200 µL) in separate tubes at 37 °C between 24 and 96 h. The control experiment did not contain the seaweed extracts. Aliquots were drawn from each tube at 48, 72 and 96 h and were viewed under a transmission electron microscope (TEM) (Carl Zeiss Libra 120 Plus [120 KV], Oberkochen, Germany).

### TEM analysis

Aβ_1-42_ and/or seaweed extracts (10 µL) from each experiment or tube were place on different copper grids coated with carbon. Solution of uranium acetate (2%) was prepared and used to stain each grid. The grids were allowed to dry and thereafter scanned with TEM. Electron micrographs and/or images obtained from each copper grid and/or experiment were observed and analysed.

### Aβ1-42 disaggregation assay

Aβ_1-42_ was prepared as described above, and 100 µL was placed in an Eppendorf tube. The tubes were incubated for 48 h to obtain preformed and/or matured amyloid fibrils. After 48 h, seaweed extracts were added to different tubes containing Aβ_1-42_ except the control. The tubes were incubated further for 96 h. Thereafter, 10 µL solution from each tube was gently placed on different copper grids and was stained with uranyl acetate (2%). The grids were allowed to dry and were scanned with TEM (Olasehinde, Olaniran, et al. 2019).

### Thioflavin-T (Th-T) assay

Aβ_1-42_ was prepared appropriately as described above, and 100 µL was placed in different tubes. After incubation at 37 °C for 48 h, seaweed extracts were added to different tubes except the control which contains Aβ_1-42_ and buffer solution. An aliquot from different tubes was placed in different wells of a black 96-well plate. Thioflavin (1 mM) was dissolved in a mixture of glycine and sodium hydroxide (pH 8.5). Thioflavin solution (40 µL) was added to the solution containing Aβ_1-42_ and/or seaweeds in the black 96-well plate and was mixed appropriately. A mixture of phosphate buffer and thioflavin solution was used as the blank. Fluorescence intensity of the thioflavin solution from each well was measured at 450 nm (excitation) and 480 nm (emission) using a microtitre plate reader (Synergy Mx Biotech, USA). Thioflavin assay was performed for 24, 48, 72 and 96 h for each tube containing Aβ_1-42_ and/or seaweed extracts (Olasehinde, Mabinya, et al. 2019).

### Statistical analysis

GraphPad prism 5.0 software was used for statistical analysis. Data obtained from the study was subjected to analysis of variance (ANOVA) and were expressed as mean ± standard error of mean (SEM). Significance difference was considered at *p* < .05.

## Results

### Phenolic constituents of seaweed extracts

**Figure 1.**
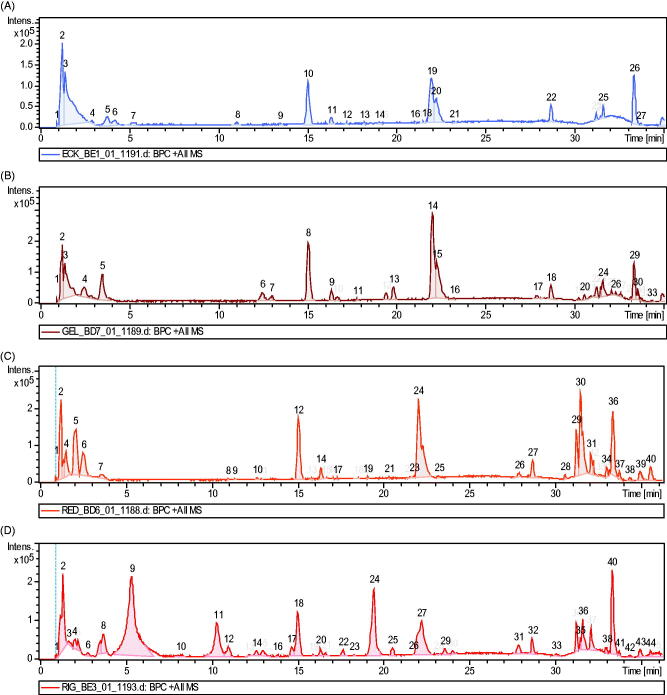
Chromatograms of seaweed extracts (A) ECK-AQ; (B) GEL-AQ; (C) RED-AQ and (D) URL-AQ.

LC-MS chromatograms revealed different peaks representing different compounds as shown in Figure 1(A-D). Phenolic compounds such as phlorotannins, flavonoids and phenolic acids were identified in the macroalgal extracts (Table 1). Phloroglucinol was the only phlorotannin identified in ECK-AQ, RED-AQ and URL-AQ but was absent in GEL-AQ (Tables 1–4). Rutinose and 1-caffeoyl-4-deoxyquinic acid as well as 3-hydroxyflavone were identified in ECK-AQ and were not present in other extracts (Table 1). Furthermore, phenolic acids such as vanillic and syringic acids were identified in GEL-AQ (Table 2). Flavonoids such as epicatechin-3 glucoside and catechin were identified in ECK-AQ and GEL-AQ (Tables 1 and 2). However, RED-AQ contains epicatechin-3 glucoside, while URL-AQ had catechin (Tables 3 and 4). Furthermore, myricetin and taxifolin that is also referred to as dihydroquercetin were present in URL-AQ but not in other seaweed extracts (Table 4). Other flavonoids present in the extracts include quercetin, 3,7-dimethyl quercetin, 5,7-dimethoxyflavone, 3,5,7-trimethylfavone and biochanin A.

**Table 1.** Phenolic constituents of aqueous extract of *Ecklonia maxima*.

Peak No	Compound	RT	[M + H]^+^ *m/z*	MS/MS fragment ions *m/z*
2	Phloroglucinol	1.32	127.02	97.05, 99.02
10	Vulgaxanthin I	15.15	340.25	100.11, 141.11
11	Epicatechin-3 glucoside	16.45	453.33	100.11, 128.06, 210.14
19	Luteoliflavan	22.04	274.27	256.25
20	(+) – Catechin	22.25	290.26	242.24
21	3-Hydroxyl flavone	23.15	239.15	123.03, 140.94
26	Biochanin A	33.32	284.29	130.12, 158.14, 284.29
27	Rutinose	34.33	326.33	186.23, 326.33
28	1-Caffeoyl-4-deoxyquinic acid	34.90	338.33	154.15, 338

RT: retention time.

**Table 2.** Phenolic constituents of aqueous extract of *Gelidium pristoides*.

Peak No	Compound	RT	[M + H] ^+^*m/z*	MS/MS fragment ions *m/z*
4	Vanillic acid	2.55	166.08	103.05
8	Vulgaxanthin I	15.07	340.25	308.24
9	Epicatechin-3 glucoside	16.45	453.33	343.23
13	Syringic acid	19.87	196.13	108.04
14	Luteoliflavan	22.03	274.27	256.25
15	Catechin	22.25	290.26	242.24
16	3,7-Dimethyl quercetin	23.23	331.24	278.21
29	Biochanin A	33.32	284.29	–
30	3,5,7-trimethoxy flavone	35.52	312.32	186.19

RT: Retention time.

**Table 3.** Phenolic constituents of aqueous extract of *Gracilaria beckeri*.

Peak No	Compound	RT	[M + H]^+^ *m/z*	MS/MS fragment ions *m/z*
2	Phloroglucinol	1.32	127.02	99.03
9	Quercetin	11.37	302.22	–
12	Vulgaxanthin I	15.09	340.25	100.11, 182.15, 340.25
14	Epicatechin-3 glucoside	16.39	453.33	343.32
23	3,7-Dihydroxy-3,4-dimethoxy	21.80	314.24	–
	Flavone			
24	Luteoliflavan	22.07	274.27	230.24
25	3,7-Dimethyl quercetin	23.23	331.24	278.21
30	5,7-Dimethoxyflavone	31.45	282.27	264.21
34	Biochanin A	33.32	284.29	–

RT: retention time.

**Table 4.** Phenolic constituents of aqueous extract of *Ulva rigida*.

Peak No	Compound	RT	[M + H]^+^ *m/z*	MS/MS fragment ions *m/z*
2	Phloroglucinol	1.32	127.02	99.01
8	Myricetin 3,7,3',4',5'- pentamethyl ether	3.74	389.18	283.12
12	Vulgaxanthin I	15.09	340.25	–
24	Taxifolin	19.49	304.08	111.00, 159.95
27	Catechin	22.25	290.26	88.07, 118.08
34	Biochanin A	33.32	284.29	–

RT: Retention time.

### Antioxidant activity of seaweed extracts

**Figure 2.**
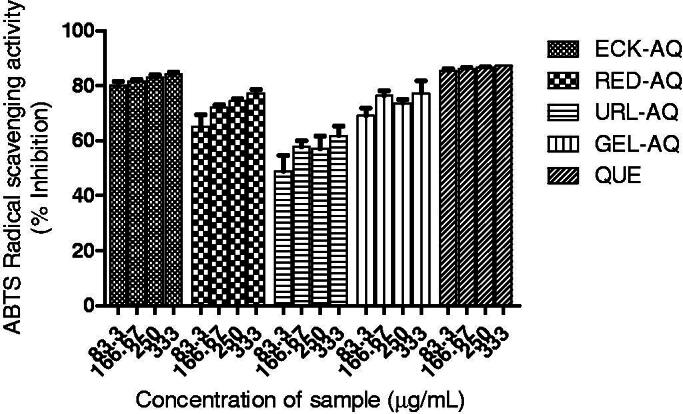
ABTS radical scavenging activities of seaweed extracts. QUE: quercetin.

**Figure 3.**
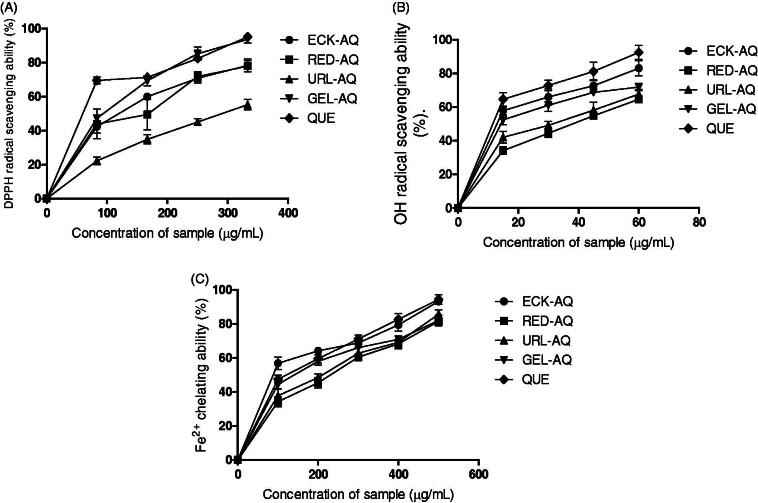
Radical scavenging and metal chelating activities of seaweed extracts. (A) DPPH radical scavenging ability (%). (B) OH radical scavenging ability (%). (C) Fe^2+^ chelating ability (%). QUE: Quercetin.

The seaweed extracts scavenged ABTS radical as shown in Figure 2. The observed scavenging activity exhibited by the extracts was above 60% at the highest concentration (333 µg/mL). URL-AQ exhibited the least activity with scavenging activity of 61.8%, while ECK-AQ showed the highest activity with 84.2% scavenging activity at the highest concentration. The activity of ECK-AQ was not significantly differently from the control (quercetin). The seaweed extracts also scavenged DPPH radicals (Figure 3(A)). URL-AQ (278.90 µg/mL) exhibited the least scavenging activity against DPPH radicals as shown by the IC_50_ values in Table 5. The scavenging activity of GEL-AQ (153.22 µg/mL) was significantly higher compared to RED-AQ (186.71 µg/mL) and ECK-AQ (181.66 µg/mL). Figure 3(B) depicts the scavenging activity of the seaweed extracts against OH radical. At the highest concentration (60 µg/mL), the seaweed extracts showed scavenging activity above 60%. ECK-AQ (30.42 µg/mL) showed significantly higher scavenging activity compared to GEL-AQ (33.70 µg/mL), URL-AQ (38.52 µg/mL) and RED-AQ (41.23 µg/mL) as revealed in Table 5. Furthermore, the seaweed extracts were able to chelate Fe^2+^ as shown in Figure 3(C).

**Table 5.** IC_50_ (µg/mL) of aqueous-ethanol extracts of some seaweeds.

Extract	ECK-AQ	RED-AQ	URL-AQ	GEL-AQ	QUE	GAL
DPPH	181.66 ± 0.36^d^	186.71 ± 6.53^d^	278.90 ± 4.51^a^	153.22 ± 1.67^e^	136.57 ± 1.22^b^	–
OH	30.42 ± 0.68^a^	41.23 ± 1.37^c^	38.52 ± 0.98^d^	33.70 ± 0.70^b^	28.69 ± 0.79^c^	–
Fe-chelation	280.47 ± 5.08^c^	334.45 ± 6.53^a^	320.92 ± 5.67^b^	303.28 ± 2.39^d^	279.66 ± 9.94^c^	
AChE	49.41 ± 2.02^a^	64.56 ± 1.17^b^	56.60 ± 4.05^c^	52.70 ± 1.85^c^	–	37.84 ± 1.03^d^
BChE	52.11 ± 2.82^a^	63.03 ± 4.80^d^	71.19 ± 2.92^b^	58.28 ± 1.79^c^	–	39.61 ± 1.08^e^

Value represent mean ± standard deviation of replicates (*n* = 3). Values with different superscript letter along the same column are significantly different (*p*<.05). – Not determined. QUE: Quercetin; GAL: Galathanmine.

The chelating activity of the extracts against Fe^2+^ increased with increase in concentration. Moreover, ECK-AQ (280.47 µg/mL) exhibited the highest chelating activity followed by GEL-AQ (303.28 µg/mL), URL-AQ (320.92 µg/mL) and RED-AQ (334.45 µg/mL) (see Table 5).

### Modulation of cholinesterase activities

**Figure 4.**
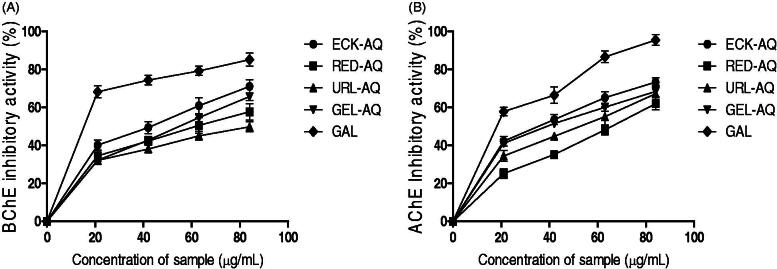
Cholinesterase inhibitory activities of seaweed extracts. (A) BChE inhibitory activity (%). (B) AChE inhibitory activity (%). GAL: Galanthamine.

The modulatory effect of the seaweed extracts on butyrylcholinesterase and acetylcholinesterase was also determined. Figure 4(A,B) show that the extracts reduced the activity of BChE and AChE, although higher inhibitory effects were observed on the latter compared to the former. At the highest concentration of the extracts, the highest BChE inhibitory activity was observed for ECK-AQ, GEL-AQ, URL-AQ and RED-AQ (Figure 4(A)). The IC_50_ values in Table 5 revealed that ECK-AQ (52.11 µg/mL) exhibited the highest inhibitory activity against BChE, while URL-AQ (71.19 µg/mL) showed the least. Similarly, the interaction of the seaweed extracts led to a significant decrease in AChE activity *in vitro* (Figure 4(B)). RED-AQ (64.56 µg/mL) exhibited significantly lower AChE inhibitory activity compared to ECK-AQ (49.41 µg/mL), GEL-AQ (52.70 µg/mL) and URL-AQ (56.60 µg/mL). Moreover, all the extracts showed potent inhibitory activity above 60% (Figure 4(B)). Galathanmine exhibited higher AChE and BChE inhibitory activities compared to the seaweed extracts.

### Modulatory effects of seaweed extracts on Aβ_1-42_ aggregation

**Figure 5.**
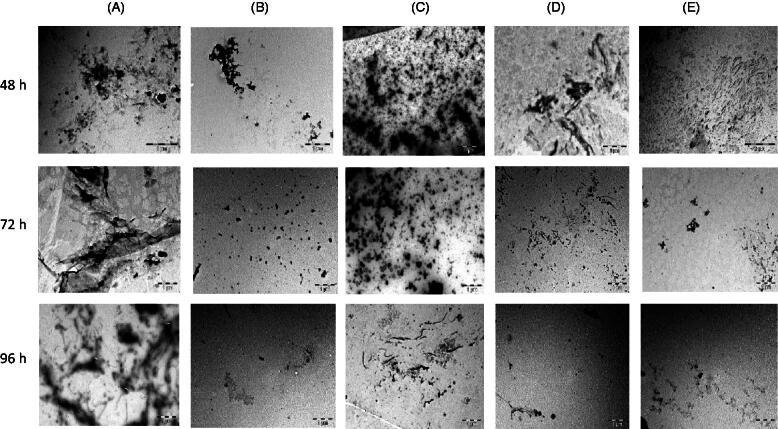
Electron micrographs showing the effects of seaweed extracts (200 µg/mL) on formation of Aβ_1-42_ fibrils and aggregation at different intervals. (A) Control (Aβ_1-42_); (B) Aβ_1-42_ + ECK-AQ; (C) Aβ_1-42_ + RED-AQ; (D) Aβ_1-42_ + URL-AQ; (E): Aβ_1-42_ + GEL-AQ.

The modulatory effect of the seaweed extracts on Aβ_1-42_ was determined using electron microscope and thioflavin-T assay. The electron micrographs in Figure 5(A) revealed the formation of matured amyloid fibrils and aggregation of the protein in the control experiment after 48 h. Moreover, continuous aggregation of Aβ_1-42_ was observed after 72 and 96 h. ECK, RED-AQ, GEL-AQ and URL-AQ were incubated separately with Aβ_1-42_ for 96 h as shown in Figure 5(B–E), respectively. Figure 5(B–E) shows that the seaweed extracts reduced and/or inhibited formation of matured fibrils and prevented continuous aggregation of the protein after incubation for 48–96 h.

### Disaggregation effects of seaweed extracts on matured Aβ_1-42_ fibrils

**Figure 6.**
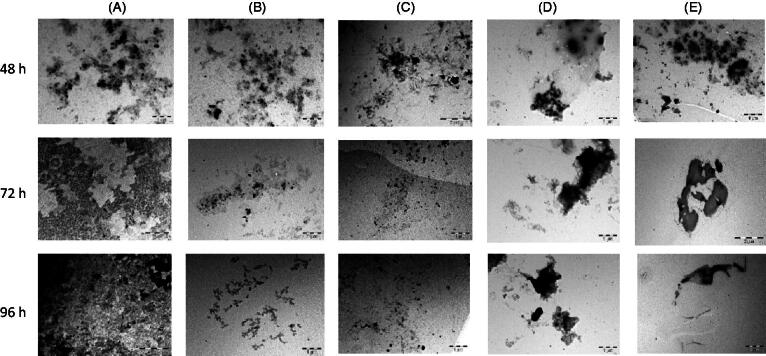
Electron micrographs showing the effects of seaweed extracts (200 µg/mL) on disaggregation of preformed Aβ_1-42_ at different intervals. (A) Control (Aβ_1-42_); (B) Aβ_1-42_ + ECK-AQ; (C) Aβ_1-42_ + RED-AQ; (D) Aβ_1-42_ + URL-AQ; (E): Aβ_1-42_ + GEL-AQ. The seaweed extracts were added to Aβ_1-42_ after 48 h.

**Figure 7.**
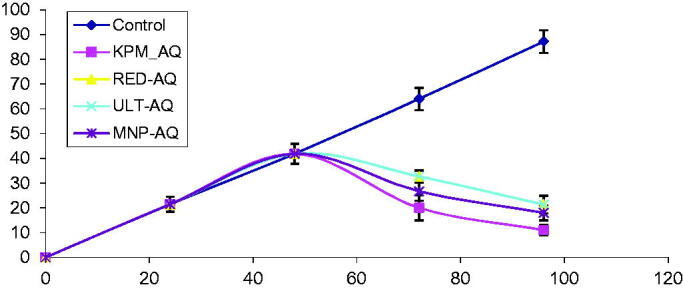
Thioflavin-T fluorescence intensity showing effects of seaweed extracts (200 µg/mL) on disaggregation of Aβ_1-42_ at different intervals. Treatment with the seaweed extracts occurred at 48 h.

To measure the disaggregation effect of the seaweed extracts, Aβ_1-42_ (without the extracts) were incubated for 48 h to obtain protein aggregates and matured amyloid fibrils. The seaweed extracts were added separately to the matured fibrils after 48 h. Figure 6(A) revealed the formation of matured fibrils and further aggregation of the protein. However, disappearance of amyloid fibrils and disaggregation of the preformed and/or matured Aβ_1-42_ fibrils was observed after 72 and 96 h in Aβ_1-42_ treated with seaweed extracts as shown in Figure 6(B–E). Figure 7 shows the results obtained from thioflavin-T assay. The result revealed that fluorescence intensity of the control increased with increase in incubation time. However, after treatment with the seaweed extracts after incubation for 24 h, fluorescence intensity reduced significantly compared to the control.

## Discussion

One of the pathological hallmarks of AD involves damage to the neurons which impairs neurotransmission and cognitive function. Free radical-induced neuronal damage is an important mechanism in the progression of AD (Zhao and Zhao 2013). The brain is susceptible to free radical attack due to low antioxidant mechanism; hence, overproduction of reactive oxygen species may cause oxidative stress-induced neuronal damage (Oboh et al. 2013). Our findings revealed that aqueous extracts from the selected seaweeds scavenged ABTS, DPPH and OH radicals and were able to chelate Fe^2+^. The observed scavenging and chelating activities suggest the antioxidant potentials of ECK-AQ, RED-AQ, URL-AQ and GEL-AQ. The antioxidant potentials of the seaweed extracts may prevent oxidative damage to neurons and improve neuronal dysfunction or neurodegeneration associated with redox imbalance. The antioxidant activities of the seaweed extracts may be linked to their bioactive constituents. Phloroglucinol, quercetin, catechin, epicatechin-3 glucoside, 3,5,7-dimethoxyflavone and biochanin A that were present in the extracts are potent antioxidants and may contribute to the observed radical and metal-chelating activities due to their ability to donate hydrogen and abstract electron (Paganga et al. 1996; Quéguineur et al. 2012; Tatsimo et al. 2012; Viau et al. 2016).

Cholinergic enzymes are very important in neurotransmission and regulate processes involved in cognitive function (Ferreira-Vieira et al. 2016). Loss of memory in AD cases has been attributed to disruption of cholinergic function via increased AChE and BChE activities as well as low levels of acetylcholine (Olasehinde et al. 2017). In this study, we evaluated the effects of some seaweed extracts on cholinesterases. Our results revealed that ECK-AQ, RED-AQ, URL-AQ and GEL-AQ inhibited acetylcholinesterase activity which is consistent with the report of Shanmuganathan and Devi (2016). Similarly, the seaweed extracts exhibited inhibitory effect on butyrylcholinesterase activities. A decrease in acetylcholinesterase and butyrylcholinesterase activities has been shown as an effective strategy to mitigate cholinergic deficit in AD (Lane et al. 2006; Oboh, Adewuni, et al. 2018). Hence, the inhibitory effects of these seaweed extracts on the cholinergic enzymes may improve cholinergic function. Our findings reveal that the extracts showed higher inhibitory effect on acetylcholinesterase compared to butyrylcholinesterase. Moreover, the active constituents of the extracts may influence their enzyme inhibitory properties. Burmaoglu et al. (2018) reported that some phloroglucinol derivatives showed potent inhibitory effect on BChE and AChE. Furthermore, the synergistic effects of other compounds (quercetin, catechin, epicatechin-3-glucoside) present in the extracts may also contribute to the observed decrease in BChE and AChE activities as their inhibitory effects on these cholinergic enzymes have been reported (Lane et al. 2004; Biradar et al. 2014; Suganthy and Devi 2016; Olasehinde, Olaniran, et al. 2019).

High cholinesterase activity in AD has been linked with the formation of neurotoxic amyloid plaques (Mushtaq et al. 2014). Carvajal and Inestrosa (2011) reported that AChE promotes the production of Aβ thereby forming complexes with the protein fibrils. Previous reports have also shown that BChE contributes to the formation Aβ plaques and neurofibrillary tangles in the neocortical region of the brain (Perry et al. 1978; Mushtaq et al. 2014). Matured Aβ fibrils form clogs around the neurons, hinder interactions between brain cells and alter cerebral brain functions (Shanmuganathan et al. 2018). Furthermore, continuous aggregation of Aβ protein leads to the formation of plaques, neuro-inflammation, neurodegeneration, cognitive dysfunction and ultimately memory loss (Mokhtar et al. 2013; Heneka et al. 2015). Recent research has been on finding natural compounds capable of inhibiting the aggregation of Aβ fibrils in Alzheimer’s disease model. Our findings revealed that ECK-AQ, RED-AQ, URL-AQ and GEL-AQ were able to inhibit fibrillogenesis and suppressed continuous aggregation of matured amyloid fibrils in a two-phase experiment. In the first phase, the seaweeds showed potent inhibition of formation and/or aggregation of Aβ_1-42_ fibrils. While Aβ_1-42_ fibrils and aggregates were formed in the control experiment, a reduction in the fibrils was observed after co-treatment with the seaweed extracts. However, more Aβ_1-42_ aggregates were observed in RED-AQ and URL-AQ incubated with Aβ_1-42_ after 48 h compared to ECK-AQ and GEL-AQ. Similar results were obtained after 72 and 96 h. Aggregation of Aβ_1-42_ was inhibited after co-treatment with the seaweed extracts as shown by the smaller fragments of peptides and/or fibrils. Larger amount of aggregates was observed after co-treatment with RED-AQ and GEL-AQ but lesser than the control. This suggests that ECK-AQ and GEL-AQ may exhibit better inhibitory effect on Aβ_1-42_ formation and aggregation compared to RED-AQ and URL-AQ. These findings correlate with the reports of Shanmuganathan et al. (2015) and Syad and Devi (2015) which revealed that *Gelidiella acerosa* (Forsskal) Feldmann et Hamel (Gelidiellaceae) and *Padino gymnospora* (Kutzing) Sonder (Dictyotaceae) extracts prevented aggregation of Aβ_25-35_, respectively. Furthermore, phloroglucinol, catechin and quercetin have been reported to inhibit β-amyloid aggregation (Vauzour 2012; Shanmuganathan et al. 2015; Yang et al. 2018). These compounds were detected in the seaweed extracts used in this study and may contribute to their anti-amyloidogenic activities.

In the second phase of the experiment, continuous aggregation of Aβ_1-42_ was observed in the control at different time intervals. Aggregation of the protein increased with time as shown in the control. However co-treatment with the extracts after 48 h led to disaggregation of the preformed and matured fibrils. The decrease in protein aggregates observed after the treatment suggests the disaggregation of Aβ_1-42_ aggregates. Better disaggregation effect of Aβ_1-42_ aggregates was exhibited by ECK-AQ and GEL-AQ compared to URL-AQ and RED-AQ. Furthermore, thioflavin assay was used in this study to quantify the levels of amyloid fibril. Thioflavin is an important quantitative marker of β-amyloid protein. Mostly, fluorescence intensity increases rapidly when thioflavin binds to amyloid fibrils. An increase in fluorescence intensity was observed in the control. Co-treatment with the seaweed extracts caused a decrease in fluorescence intensity. The observed decrease in fluorescence intensity with increase in incubation time which was exhibited by ECK-AQ, GEL-AQ, ULT and RED-AQ suggests loss or low levels of matured amyloid fibrils. This result correlates with the decrease in protein aggregates observed in the electron micrographs. The electron micrographs revealed that the extracts disassembled preformed aggregates and/or matured amyloid fibrils after 72 h. Our findings revealed that the constituents of the extracts may contribute to the inhibition of aggregation and disaggregation of Aβ_1-42_ fibrils.

## Conclusion

This study reveals the phenolic composition, antioxidant, cholinesterase inhibitory and anti-amyloidogenic activities of aqueous extracts of *G. beckeri, G. pristoides*, *U. rigida* and *E. maxima*, via their radical scavenging and metal-chelating activities, acetylcholinesterase and butyrylcholinesterase activities, as well as inhibition of Aβ_1-42_ aggregation and disaggregation of matured amyloid fibrils. Phloroglucinol, catechin, epicatechin-3 glucoside, quercetin, kaempferol and 3,5,7-trimethoxy flavone were present in the extracts. Our findings suggest potential neuroprotective effects of these extracts which could be linked to the presence of some phenolic compounds. Hence, these seaweeds may be good sources of antioxidants, cholinesterase and β-amyloid aggregation inhibitors and could be explored as an alternative therapeutic strategy for the management of AD.
